# One Health for all: Implementing international frameworks with local communities

**DOI:** 10.1371/journal.pgph.0005520

**Published:** 2025-12-11

**Authors:** Mélodie Ruwet, Michelle Rourke, Kaosar Afsana, Fatimah Ahamad, Elva Borja, Kelley Lee, Clare Wenham, Naomi Woyengu, Sara E. Davies

**Affiliations:** 1 Griffith Asia Institute, Griffith University, Brisbane, Australia; 2 Griffith Law School, Griffith University, Brisbane, Australia; 3 James P Grant School of Public Health, BRAC University, Dhaka, Bangladesh; 4 Sunway Centre for Planetary Health, Sunway University, Selangor, Malaysia; 5 Veilomani One Health Group/VET Essentials, Suva, Fiji; 6 Faculty of Health Sciences, Simon Fraser University, Burnaby, Canada; 7 Department of Health Policy, London School of Economics, London, United Kingdom; 8 Hauskuk Initiative, Madang, Papua New Guinea; 9 Griffith Asia Institute, Griffith University, Brisbane, Australia; PLOS: Public Library of Science, UNITED STATES OF AMERICA

## Introduction

The One Health concept emphasizes the interdependence of human, animal and environmental health. While the term “One Health” has only gained traction in this century, the idea itself is much older. For instance, many Indigenous Peoples and local communities have traditional cosmologies that recognize the interconnectedness of the ecosystem and the role of humans within it [1, 2]. It is the industrialized-focused worldview that has only recently come to terms with the fact that humans are part of a complex global ecology, not the masters of it. While some progress has been made in recognizing the importance of local, traditional and Indigenous knowledges in One Health policy documents, and towards including gender equality, disability and social inclusion (GEDSI) considerations, hurdles remain to meaningfully incorporate context-specific knowledge in practice (see S1 Fig for definitions of the different types of knowledges).

In this article, we advocate for funding and engagement in deep context-specific social research before funding and engaging in One Health interventions. Through our own work on the Indo-Pacific Initiative for Sustainable Animal Health Cooperation, we seek to understand how factors such as gender and social inclusion can inform the uptake or rejection of One Health practices within local communities in the region. To keep communities safe and ensure equitable health outcomes, it is worth the time, money and effort to understand the dynamics that shape and motivate local interactions between humans, animals and the environment. This will undoubtedly help to develop more tailored, appropriate, trusted and sustainable One Health interventions.

### Community-level knowledge in One Health policy

The One Health Joint Plan of Action 2022–2026 [3] (OH JPA) is an example of a recent international framework that is seeking to address health challenges including zoonotic spillovers, through supporting policy and research coordination and action. The highest international authorities have agreed to the plan: the Food and Agriculture Organization of the United Nations, the World Organization for Animal Health, the World Health Organization and the United Nations Environment Programme (the “Quadripartite” organizations). The OH JPA is not a binding document, but a normative one. It sets out the broadly agreed standards and practices on how to prevent some of the biggest health threats of our time, with reference to implementation at the international, regional and national levels. The OH JPA recognizes the importance of involving local communities in One Health interventions and including traditional knowledge “in tandem with scientific knowledge” (p. 20), but the implementation of One Health at the community level is, by necessity, lacking in detail and left to national authorities. How might implementing parties provide meaningful, systematic inclusion when implementing One Health action locally, and how might researchers and practitioners support cooperative inquiry and action?

One of the three “pathways to change” outlined by the OH JPA is the strengthening of data, evidence and knowledge for “technical tools, protocols and guidelines, information and surveillance systems” (p. 15). This pathway to change is associated with 6 “action tracks” to help prevent, predict, detect and respond to health threats around the world.

The “action tracks”, as their names suggest, are about actively changing allocation of resources and priorities, particularly regarding zoonotic spillovers (action track 2 – “reducing the risks from emerging and re-emerging zoonotic epidemics and pandemics”). Some items are normative and many place clear emphasis on intervention, while leaving countries the space to implement according to their own resources. This makes sense given different capacities and risks at the country level. However, as the action tracks pertain to community-level implementation, the bias towards action itself can imply that local actors are the source of risky behaviors who require education about risk mitigation strategies (see, e.g., 2.2.5 and 2.2.6), rather than knowledgeable and experienced agents that employ varied strategies to keep their environment, animals and communities healthy across generations. The overarching idea communicated by this “action bias” (see Patt and Zeckhauser, 2000) [4] is that if local communities are made aware of the importance of One Health, and then taught risk mitigation strategies, spillover risks will be eliminated.

### Why local, traditional and Indigenous knowledges?

The OH JPA acknowledges the existence of local, traditional and Indigenous knowledge, all framed as a complement to the “scientific knowledge” to be introduced to the community (p. 20). Community knowledge is not an “extra” add on, it is essential for community engagement and buy-in. A deeper, context specific approach would be to treat One Health implementation at the community level as a co-design partnership, with local, traditional and Indigenous knowledges forming the very basis of any One Health interventions. Without understanding of local contexts, top-down interventions miss the opportunity to identify essential community-level knowledge as the baseline for supporting sustainable practices over time and generations [5]. Action bias means there is sometimes a rush to introduce new knowledge and practices without considering the importance of conserving or enhancing *existing* biosecurity knowledge and practices [4]. It denies the wealth of knowledge that local communities can offer to One Health, and their interest in taking part in prevention activities.

It is vital to consider the incentives that drive existing behaviors and could affect the adoption of new ones. For instance, while farmers in low and middle income countries are often framed as driving spillover risk due to lack of knowledge and education, evidence points towards farmers’ decisions being rooted in structural constraints (including lack of resources and time) rather than a lack of knowledge and interest [6]. Moreover, absence of biosecurity knowledge and practices that are specifically framed as “One Health” should not be interpreted as absence of alternative ways of knowing and doing [7] that may actually be compatible with the One Health concept. Furthermore, we should not discount the fact that sometimes “best practice” in one location will not translate to another and may even do more harm than good [8]. Knowledge, Attitudes and Practices (KAP) surveys can give insights about what local stakeholders know about a certain issue and how they act. However, these surveys are not designed to inquire how gender, economic, social, cultural, and political contexts may inform local participants responses in such surveys [7]. While co-design and participatory methods are gaining traction, exploratory research that includes diverse and context-specific knowledges should equally inform the starting-point from which to design all One Health interventions at the local level.

### How to (re)define One Health: final considerations

In sum, we recommend One Health action research embrace co-design partnering approaches with communities that are context specific (gender, social, political, economic) and deeply engage with local, traditional and Indigenous knowledges (for an example of such an approach see Laalaai-Tausa et al, [9]). Aside from recognizing the plurality of epistemologies, interventions can benefit from meaningful local exchange and engagement. To achieve this, One Health research must embrace interdisciplinary approaches. There is a current reliance on veterinary science, animal health, epidemiology and related disciplines, with insights derived from the social sciences still limited. As discussed by Hinchliffe and others, many social science disciplines, including economics, geography, and political economics (but also political science) often remain absent from studies on zoonotic risks at the human-animal interface. Social science can reframe our understanding of local community as prevention champions in One Health, reframing them as risk mitigation actors rather than risk amplifiers.

## Supporting information

S1 Fig Local, Traditional, and Indigenous Knowledges. (TIFF)
