# Supplementary figures and images for: One Health for all: Implementing international frameworks with local communities
Source: PLOS Glob Public Health. 2025 Dec 11;5(12):e0005520. doi: 10.1371/journal.pgph.0005520 (PMC12697943; doi:10.1371/journal.pgph.0005520)

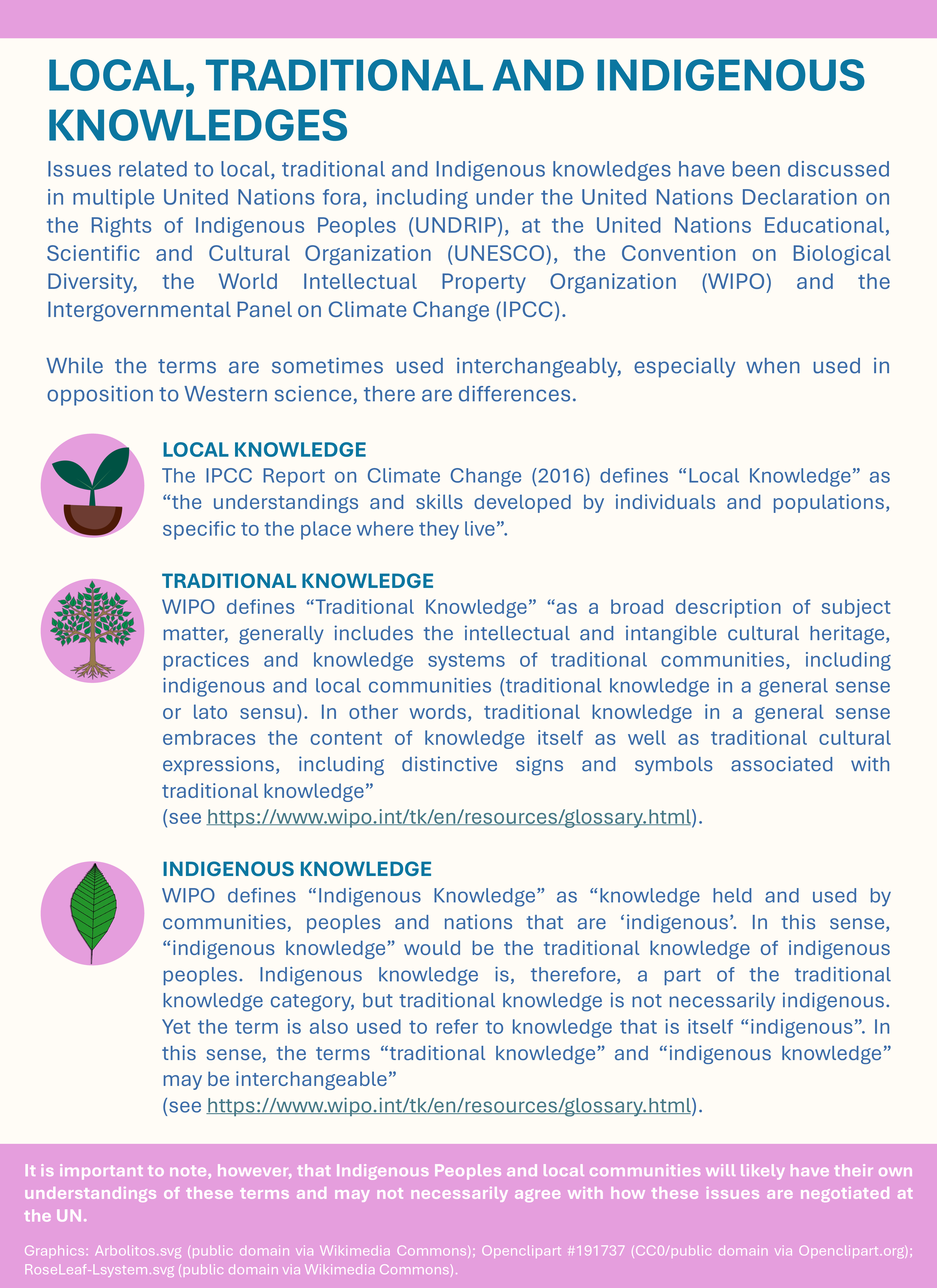

Supplement: S1 Fig — (TIFF) [file pgph.0005520.s001.tiff]
